# Contagion in Global Stock Markets during the COVID‐19 Crisis

**DOI:** 10.1002/gch2.202000130

**Published:** 2021-07-15

**Authors:** Shenze Fu, Chengkun Liu, Xinyang Wei

**Affiliations:** ^1^ School of Business Macau University of Science and Technology Taipa Macau China

**Keywords:** COVID‐19, financial contagion, stock markets

## Abstract

The impact of the coronavirus disease (COVID‐19) outbreak on global stock markets is investigated by analyzing the impact of the COVID‐19 pandemic on the stock markets of 15 countries selected from Asia, Europe, Latin America, and North America. Using extremal dependence tests of contagion, it is found that contagion effects are widespread to global equity markets in four regions. Latin America and North America are highly exposed to contagion risks, followed by Europe, with Asia being least vulnerable. Based on the time window of the crisis severity index, it is found that Latin America is most likely to be affected. The results confirm that for countries with more severe epidemics, there are stronger contagion effects. Therefore, for the governing authorities of various countries, if they want to prevent the contagion of financial crises during the pandemic, strong and timely epidemic prevention measures are very necessary.

## Graphical abstract

The impact of the COVID‐19 outbreak on global stock markets is investigated by analyzing the impact of the COVID‐19 pandemic on the stock markets of 15 countries selected from Asia, Europe, Latin America, and North America. Latin America and North America are highly exposed to contagion risks, followed by Europe, with Asia being least vulnerable.

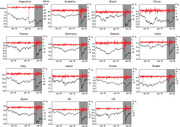

## 1 Introduction

The recent outbreak of the coronavirus disease COVID‐19 has brought about a renewed interest in financial contagion. With the increase in number of COVID‐19 cases and the absence of an effective vaccine, global investors have taken a negative outlook on the global economy and liquidated their financial market positions, affecting stock markets globally. In late March 2020, the European, German, and United States stock indices posted their sharpest falls of more than 10%, causing the most widespread global and economic disruption since the Great Depression of the 1930s.

Some scholars have investigated the effects of Covid‐19 on financial markets, including gold and oil markets, Corporate Social Responsibility (CSR), as well as Environmental, Social, and Governance. While gold markets’ efficiency is affected by the pandemic,^[^ ^1^ ^]^ gold is still a safe haven asset for the stock markets^[^ ^2^ ^]^ as well as the oil price risks.^[^ ^3^ ^]^ As to the oil market, the volatility of oil price increased following the commencement of COVID‐19^[^ ^4^ ^]^ and financial industries responded negatively to positive oil price shocks.^[^ ^5^ ^]^ During the pandemic, both oil and stock markets may encounter stronger impacts of their own and cross shocks.^[^ ^6^ ^]^ At the firm level, the Covid‐19 pandemic provides enterprises the opportunity to undertake more authentic and genuine CSR^[^ ^7^ ^]^ and relevant activities can help hospitality companies enhance their stock returns and gain stakeholders’ attention during the pandemic.^[^ ^8^ ^]^ However, exchange traded funds with higher degrees of sustainability performance can still not protect investors from financial losses in the event of the Covid‐19 pandemic.^[^ ^9^ ^]^

Although a large number of literature have confirmed the existence of financial contagion in previous financial crises, there is not much literature on the contagion of financial markets during the Covid‐19 pandemic. However, the global spread of the virus has affected different economies and their stock markets simultaneously and researchers have found significant financial contagion during COVID‐19 crisis.^[^ ^10^ ^]^ Therefore, this paper aims to investigate the contagion effect of the COVID‐19 outbreak on global stock markets by analyzing the impact of the COVID‐19 pandemic on the stock markets of 15 countries selected from Asia, Europe, Latin America, and North America. Since the sample includes not only developed countries in Europe and America but also developing countries, this article has high empirical value for reviewing the contagion effect of the Covid‐19 pandemic. While some researchers adopt the correlation between markets beyond economic fundamentals as the definition of financial contagion,^[^ ^11^, ^12^ ^]^ the extremal dependence tests selected by this paper uses the very restrictive definition that cross‐country correlations significantly increase during crisis periods.^[^ ^13^ ^]^

Several models have been employed to investigate crisis and market contagion, including the extreme value model,^[^ ^14^ ^]^ bivariate generalized autoregressive conditional heteroscedasticity,^[^ ^15^ ^]^ the Copula family of models,^[^ ^16^ ^]^ regime switching models,^[^ ^17^ ^]^ and exponential normal distribution family of models.^[^ ^13^ ^]^ While these models have advantages, they are inappropriate under the traditional mean‐variance framework of normality assumption. Rather than using linear dependence to model contagion, nonlinear models, such as the coexceedance approach,^[^ ^18^ ^]^ threshold tests,^[^ ^19^ ^]^ asymmetric dependence tests,^[^ ^20^ ^]^ and extremal dependence tests,^[^ ^21^ ^]^ are used to capture the statistical properties of asymmetric and fat‐tailed behaviors of asset returns. As some researchers have documented, extremal dependence (measured by cokurtosis and covolatility) can capture a higher number of comovements than can linear dependence for serious crises,^[^ ^22^ ^]^ this paper adopt the extremal dependence approach to test for the contagion effects of the COVID‐19 pandemic.

The contributions of this paper are as follows: First, nonlinear dependence tests of contagion are adopted to identify how Covid‐19 affects global stock markets, including cross‐country mean and skewness (cokurtosis) and cross‐country volatilities (covolatility). Second, from a global perspective, this paper fills the research gap by studying the contagion impact of the epidemic in both developed and developing countries. Third, rolling contagion tests are performed for robustness checks and the results are also compared with the traditional exponential generalized auto regressive conditional heteroskedasticity (EGARCH) conditional variance time series model.

We find that contagion effects are widespread to global equity markets in four regions during the COVID‐19 period. Latin America and North America are highly exposed to contagion risks, followed by Europe, with Asia being the least vulnerable. However, Japan is not affected by COVID‐19 through the cokurtosis and covolatility channels. Based on the time window of the crisis severity index, we found that Latin America is most likely to be affected. The results are robust when using local currencies rather than common currency denominated returns.

The paper is structured as follows. Section 2 introduces the contagion tests. Section 3 introduces the data and discusses the descriptive statistics. Section 4 discusses the empirical results. Section 5 concludes the paper.

## 2 Econometric Methodology

In order to study the contagion effect of the COVID‐19 outbreak on global stock markets, this paper first adopts extremal dependence tests of contagion in Section 2.1. These tests use higher order comoments including cokurtosis and covolatility to measure changes in relationships between assets markets, which can better characterize the asymmetric and fat‐tailed characteristics of asset returns.^[^ ^22^ ^]^ While the Section 2.1 belongs to static analysis of contagion effect, Section 2.2 conducts the dynamic analysis of contagion effect by calculating the crisis severity index using the rolling 30 days window of returns through the entire COVID‐19 period. As a comparison, the Section 2.3 uses lower order moment model EGARCH to study the contagion effect.

### 2.1 Extremal Dependence Tests

Let *r*_*i*,*t*_ and *r*_*j*,*t*_ be the asset returns of markets *i* and *j* at time *t* with mean μ and variance σ. Following previous literature,^[^ ^21^, ^23^ ^]^ cokurtosis in the precrisis (*x*) and crisis (*y*) periods are

(1) $$\begin{array}{c} {\hat{\psi }}_{k}\;\left(r_{i},r_{j}^{3}\right)=\frac{1}{T_{k}}\;\sum_{t\;=\;1}^{T_{k}}\left(\frac{r_{i,t}-{\hat{\mu }}_{i,k}}{{\hat{\sigma }}_{i,k}}\right){\left(\frac{r_{j,t}-{\hat{\mu }}_{j,k}}{{\hat{\sigma }}_{j,k}}\right)}^{3},k\;=\;x,y \end{array}$$

(2) $$\begin{array}{c} {\hat{\psi }}_{k}\;\left(r_{i}^{3},r_{j}\right)=\frac{1}{T_{k}}\;\sum_{t\;=\;1}^{T_{k}}{\left(\frac{r_{i,t}-{\hat{\mu }}_{i,k}}{{\hat{\sigma }}_{i,k}}\right)}^{3}\;\;\left(\frac{r_{j,t}-{\hat{\mu }}_{j,k}}{{\hat{\sigma }}_{j,k}}\right),k\;=\;x,y \end{array}$$

and the corresponding covolatility is

(3) $$\begin{array}{c} {\hat{\psi }}_{k}\;\left(r_{i}^{2},r_{j}^{2}\right)=\frac{1}{T_{k}}\;\sum_{t\;=\;1}^{T_{k}}{\left(\frac{r_{i,t}-{\hat{\mu }}_{i,k}}{{\hat{\sigma }}_{i,k}}\right)}^{2}\;\;{\left(\frac{r_{j,t}-{\hat{\mu }}_{j,k}}{{\hat{\sigma }}_{j,k}}\right)}^{2},k\;=\;x,y \end{array}$$

Researchers have proposed cokurtosis and covolatility tests for contagion, defined as significant changes in cokurtosis and covolatility during the crisis period compared with the precrisis period.^[^ ^21^ ^]^ The cokurtosis contagion tests from returns of market *i* to skewness of market *j*, ${\text{CK}}_{13}(i\to j;r_{i}^{1},r_{j}^{3})$, and the cokurtosis contagion test from the skewness of market *i* to returns of market *j*, ${\text{CK}}_{31}(i\to j;r_{i}^{3},r_{j}^{1})$, are given by

(4) $$\begin{array}{c} {\text{CK}}_{13}\;\left(i\to j;r_{i}^{1},r_{j}^{3}\right)=\frac{{\left({\hat{\varphi }}_{y}\left(r_{i},r_{j}^{3}\right)-{\hat{\varphi }}_{x}\left(r_{i},r_{j}^{3}\right)\right)}^{2}}{\frac{18{\hat{v}}_{y|x_{i}}^{2}+6}{T_{y}}+\frac{18{\hat{\rho }}_{x}^{2}+6}{T_{x}}} \end{array}$$

(5) $$\begin{array}{c} {\text{CK}}_{31}\;\left(i\to j;r_{i}^{3},r_{j}^{1}\right)=\frac{{\left({\hat{\varphi }}_{y}\left(r_{i}^{3},r_{j}\right)-{\hat{\varphi }}_{x}\left(r_{i}^{3},r_{j}\right)\right)}^{2}}{\frac{18{\hat{v}}_{y|x_{i}}^{2}+6}{T_{y}}+\frac{18{\hat{\rho }}_{x}^{2}+6}{T_{x}}} \end{array}sw$$

where

(6) $$\begin{array}{c} {\hat{\varphi }}_{x}\;\left(r_{i}^{a},r_{j}^{b}\right)={\hat{\psi }}_{x}\;\left(r_{i}^{a},r_{j}^{b}\right)-3{\hat{\rho }}_{x} \end{array}$$

(7) $$\begin{array}{c} {\hat{\varphi }}_{y}\;\left(r_{i}^{a},r_{j}^{b}\right)={\hat{\psi }}_{y}\;\left(r_{i}^{a},r_{j}^{b}\right)-3{\hat{v}}_{y|x_{i}} \end{array}$$

(8) $$\begin{array}{c} {\hat{v}}_{y|x_{i}}=\frac{{\hat{\rho }}_{y}}{\sqrt{1+\delta \left(1-{\hat{\rho }}_{y}^{2}\right)}} \end{array}$$

The conditional correlation coefficient during the crisis period is ${\hat{v}}_{y|x_{i}}$, where $\delta \;=\;(\sigma_{i,y}^{2}-\sigma_{i,x}^{2})/\sigma_{i,x}^{2}$ and ${\hat{\rho }}_{x}$ is the correlation coefficient in the precrisis period. *T_x_* and *T_y_* are the sample sizes for each period.

The covolatility contagion test from return volatility of market *i* to volatility of market *j* gives

(9) $$\begin{array}{c} {\text{CV}}_{22}\left(i\to j;r_{i}^{2},r_{j}^{2}\right)\;=\frac{{\left({\hat{\varphi }}_{y}\left(r_{i}^{2},r_{j}^{2}\right)-{\hat{\varphi }}_{x}\left(r_{i}^{2},r_{j}^{2}\right)\right)}^{2}}{\frac{4{\hat{v}}_{y|x_{i}}^{4}+16{\hat{v}}_{y|x_{i}}^{2}+4}{T_{y}}+\frac{4{\hat{\rho }}_{x}^{4}+16{\hat{\rho }}_{x}^{2}+4}{T_{x}}} \end{array}$$

where

(10) $$\begin{array}{c} {\hat{\varphi }}_{x}\;\left(r_{i}^{2},r_{j}^{2}\right)=\frac{1}{T_{x}}\;\sum_{t\;=\;1}^{T_{x}}{\left(\frac{r_{i,t}-{\hat{\mu }}_{i,x}}{{\hat{\sigma }}_{i,x}}\right)}^{2}\;\;{\left(\frac{r_{j,t}-{\hat{\mu }}_{j,x}}{{\hat{\sigma }}_{j,x}}\right)}^{2}-\left(1+{\hat{\rho }}_{x}^{2}\right) \end{array}$$

(11) $$\begin{array}{c} {\hat{\varphi }}_{y}\;\left(r_{i}^{2},r_{j}^{2}\right)=\frac{1}{T_{y}}\;\sum_{t\;=\;1}^{T_{y}}{\left(\frac{r_{i,t}-{\hat{\mu }}_{i,y}}{{\hat{\sigma }}_{i,y}}\right)}^{2}\;\;{\left(\frac{r_{j,t}-{\hat{\mu }}_{j,y}}{{\hat{\sigma }}_{j,y}}\right)}^{2}-\left(1+2{\hat{v}}_{y|x_{i}}^{2}\right) \end{array}$$

Under the null hypothesis, the test statistics are asymptotically distributed as ${\text{CK}}_{13},{\text{CK}}_{31},{\text{CV}}_{22}\overset{d}{\to }\chi_{1}^{2}$.

### 2.2 Crisis Severity Index

The degree of sensitivity of a market to the COVID‐19 pandemic can be determined by calculating the crisis severity index.^[^ ^24^ ^]^ The index is constructed based on the given precrisis period. The contagion tests in Equations (4), (5), and (9) are calculated using the rolling 30 days window of returns through the entire COVID‐19 period. Taking the cokurtosis test as an example, an indicator variable is constructed for each recipient sector *j*, which takes a value of 1 if the test statistic (CK_13(*i* →*j*), *j*, *t*_) in Equation (4) is significant at the 0.05 level of significance

(12) $$\begin{array}{c} I_{{\text{CK}}_{13}\left(i\to j\right),j,t}=\{\begin{array}{c} 1:\\ 0: \end{array}\begin{array}{c} {\text{CK}}_{13}\left(i\to j;r_{i}^{1},r_{j}^{3}\right)\geq 3.84\\ \text{otherwise} \end{array},j\neq i \end{array}$$

then for the second form of cokurtosis CK_31(*i* →*j*), *j*, *t*_ and covolatility $I_{{\text{CV}}_{22}(i\to j),j,t}$, the crisis severity index for the market *j* (CI_*j*,*t*_(*i* → *j*)) at time *t* is constructed by combining three channels, giving

(13) $$\begin{array}{c} {\text{SI}}_{j,t}\;\left(i\to j\right)=\;100\left(\frac{I_{{\text{CK}}_{13}\left(i\to j\right),j,t}+{\text{CK}}_{31\left(i\to j\right),j,t}+I_{{\text{CV}}_{22}\left(i\to j\right),j,t}}{3}\right) \end{array}$$

The mean of the crisis severity index for market *j* is calculated as $\mu_{{\text{SI}}_{j,t}}={\text{SI}}_{j,t}\;(i\to j)/(T_{y}-30)$, where *T_y_* is the sample size for the COVID‐19 period.

### 2.3 EGARCH Model

In order to compare the contagion tests with the traditional time series model, this paper also adopts the EGARCH conditional variance model^[^ ^25^, ^26^ ^]^ and plots the conditional volatility of each index. The model is given by

(14) $$\begin{array}{c} r_{jt}=\mu_{j}\;+b_{1}r_{it}+b_{2}r_{it}D_{\text{Crisis}\;}+u_{jt} \end{array}$$

(15) $$\begin{array}{c} h_{jt}=\;\exp(c+\theta z_{jt-1}+\gamma \left|z_{jt-1}\right|-E\left(\left|z_{jt-1}\right|\right)+\delta \log\left(h_{jt-1}\right)+d_{1}r_{it-1}^{2}+d_{2}r_{it-1}^{2}D_{\text{Crisis},t-1} \end{array}$$

Equation (14) is the mean equation. *D*_Crisis_ is a dummy variable that is equal to 1 if in the crisis period and 0 otherwise. *R_jt_* is the return of the market *j*. The normal effect of shocks from the market *i* to the market *j* is controlled by the parameter *b*_1_, which evaluates the comovement of the two markets as a result of common shocks. The parameter *b*_2_ shows if there is a contagion effect in mean, which is beyond the normal effect in a given crisis period. The parameter *b*_2_ evaluates the contagion in volatility by studying how the comovement changed during the crisis period. Equation (15) is the variance equation and *h* *_jt_* is the conditional variance. In addition to the usual regression items in the EGARCH model, the parameter *d*_1_ controls the normal volatility spillover from the market *i* while the parameter *d*_2_ reflects the effect of shocks beyond the normal volatility spillover during the crisis period.

## 3 Data and Descriptive Statistics

**Figure 1:**
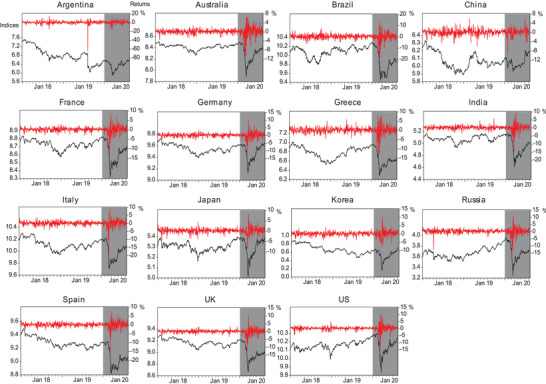
Daily indices and returns of equity markets for 15 countries from January 2, 2018 to August 17, 2020. Note: Shaded areas denote the COVID‐19 period.

We analyze the impact of the COVID‐19 pandemic on the stock markets of 15 countries selected from Asia, Europe, Latin America, and North America.^[^ ^27^ ^]^ Daily percentage equity returns are calculated by *r_t_* = 100ln(*P_t_*/*P*_*t* − 1_), where *P_t_* is the daily closing price at time *t*. Our sample covers the period January 1, 2018 to August 17, 2020. We begin in 2018 to avoid the collapse of the oil excess capacity bubble in China.^[^ ^28^ ^]^ The entire sample period is divided into two sub‐periods: the precrisis period is January 1, 2018 to December 30, 2019 ( *T_x_* = 518 observations) and the COVID‐19 period is December 31, 2019 to August 17, 2020 (*T_y_* = 165 observations). This paper is following the literature by choosing December 31, 2019, when China reported the first case of COVID‐19 to the World Health Organization (WHO), as the starting date for the COVID‐19 pandemic.^[^ ^10^, ^29^ ^]^ **Figure** **1** shows the daily prices (left axis) and returns (right axis) of global equity markets during the period 2018–2020. As expected, the stock prices demonstrate an overall downward trend, dropping to their lowest points in March 2020 for all countries, and stock returns show dramatic volatility, clustering over the period of the COVID‐19 pandemic.

**Table** **1** shows the extremal dependences between China and selected stock markets for the two periods. We find that the stock markets of most countries are more highly correlated with the Chinese stock market during the COVID‐19 period than in the precrisis period. The higher values of cokurtosis and covolatility during the COVID‐19 period indicate a greater number of outliers located in two tails of joint distribution, implying a high contagion risk.

**Table 1:** Summary of fourth‐order comoment returns between the Chinese and 14 equity markets for the precrisis and COVID‐19 periods

Country^a)^ (*j*)	Precrisis period			COVID‐19 period		
	${\hat{\psi }}_{x}(r_{i},r_{j}^{3})$	${\hat{\psi }}_{x}(r_{i}^{3},r_{j})$	${\hat{\psi }}_{x}(r_{i}^{2},r_{j}^{2})$	${\hat{\psi }}_{y}(r_{i},r_{j}^{3})$	${\hat{\psi }}_{y}(r_{i}^{3},r_{j})$	${\hat{\psi }}_{y}(r_{i}^{2},r_{j}^{2})$
Asia						
Australia	1.684	2.092	1.741	3.019	1.935	1.835
China	6.104	6.104	6.104	12.312	12.312	12.312
India	0.724	1.275	1.181	4.474	2.229	2.236
Japan	2.027	1.796	1.901	1.831	1.935	1.067
Korea	2.324	2.384	2.133	2.609	1.238	1.541
Europe						
France	1.290	1.925	1.464	3.458	1.182	1.670
Germany	1.259	1.840	1.461	3.598	1.226	1.677
Greece	1.386	1.849	1.629	3.410	1.575	1.959
Italy	0.784	1.808	1.445	5.404	0.762	1.963
Russia	0.260	0.891	0.969	1.209	0.812	1.372
Spain	0.872	1.463	1.198	3.930	1.178	1.778
United Kingdom	1.598	1.745	1.682	3.722	1.922	1.829
Latin America						
Argentina	−9.217	0.040	1.162	2.784	0.235	2.057
Brazil	−0.123	0.159	1.424	3.281	0.466	1.884
North America						
United States	0.511	0.991	1.223	3.265	0.951	1.835

^a)^ Statistics are measured as follows: cokurtosis_13_ $\left({\hat{\psi }}_{k}(r_{i},r_{j}^{3})\right)$: returns of market *j* and cubed returns of the Chinese equity market in Equation (1); cokurtosis_31_ $\left({\hat{\psi }}_{k}(r_{i}^{3},r_{j})\right)$: cubed returns of market *j* and returns of the Chinese equity markets in Equation (2); covolatility_22_ $\left({\hat{\psi }}_{k}(r_{i}^{2},r_{j}^{2})\right)$: squared returns of market *j* and squared returns of the Chinese equity markets in Equation (3).

## 4 Empirical Analysis

Prior to computing the statistics for the contagion tests, we follow existing literature^[^ ^20^, ^24^ ^]^ and use a 15‐variate vector autoregression model with two lags to control for market fundamentals and filter out possible serial autocorrelations in stock market returns between the market *i* (China) and each *j* country.^[^ ^30^ ^]^

**Table** **2** presents the empirical results of contagion tests based on changes in cokurtosis and covolatility during the COVID‐19 period. The results show significant widespread contagion effects from China's equity market to selected equity markets in four regions during the COVID‐19 period, either through the cokurtosis or covolatility channels. Among the four regions, Latin America and North America are highly exposed to contagion risk, followed by Europe, with Asia being the least vulnerable, evidenced by both cokurtosis channels being in operation. Japan is not affected by COVID‐19 through the cokurtosis and covolatility channels. Given that the empirical results of contagion tests are conditional on the choice of crisis and noncrisis dates, rolling contagion tests are performed for robustness checks.^[^ ^31^ ^]^

**Table 2:** Test statistics for contagion based on changes in cokurtosis and covolatility during the COVID‐19 pandemic using common currency denominated returns (US dollars)

Country^a)^ (*j*)	Contagion tests					
	CK_13_	*p*	CK_31_	*p*	CV_22_	*p*
Asia						
Australia	5.42	0.02**	5.19	0.02**	3.78	0.05**
India	35.50	0.00**	0.89	0.34	0.06	0.80
Japan	0.01	0.93	1.22	0.27	1.49	0.22
Korea	0.00	0.97	20.53	0.00**	1.28	0.26
Europe						
France	8.21	0.00**	20.27	0.00**	0.15	0.70
Germany	4.67	0.03**	17.77	0.00**	1.24	0.27
Greece	9.53	0.00**	3.71	0.05**	2.19	0.14
Italy	71.90	0.00**	42.94	0.00**	0.15	0.70
Russia	5.17	0.02**	4.78	0.03**	1.43	0.23
Spain	19.16	0.00**	12.34	0.00**	0.29	0.59
UK	5.09	0.02**	1.13	0.29	1.48	0.22
Latin America						
Argentina	1304.96	0.00**	7.78	0.01**	25.70	0.00**
Brazil	55.87	0.00**	3.44	0.06*	1.30	0.25
North America						
US	19.43	0.00**	6.83	0.01**	0.13	0.72

^a)^ CK_13_: cokurtosis contagion test in Equation (4); CK_31_: cokurtosis contagion test in Equation (5); CV_22_: covolatility contagion test in Equation (9). *p = 0.1, **p = 0.05.

**Figure 2:**
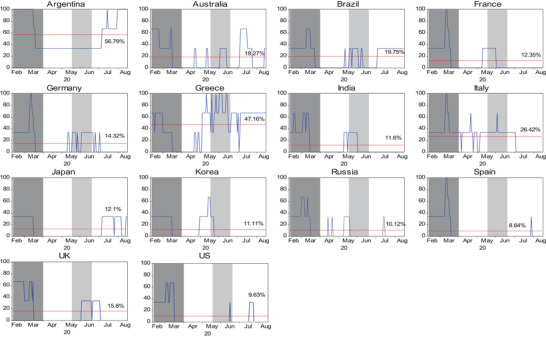
Crisis severity index for 14 countries during the COVID‐19 period using common currency denominated returns (US dollars). Note: Crisis severity index (SI_*j*,*t*_(*i* → *j*)) is calculated in Equation (13) and unit is percentage (%). The horizontal line represents the mean of crisis severity index ($\mu_{{\text{SI}}_{j,t}}$).

To evaluate the markets more likely to be affected by the COVID‐19 crisis, the crisis severity index is calculated and presented in **Figure** **2**. Overall, COVID‐19 is likely to affect a large number of countries in four regions, especially in Latin America, as indicated by 56.79% for Argentina and 19.75% for Brazil. This is not surprising given that most of these countries were seriously affected by COVID‐19 in the first 3 months of the pandemic (shaded area). In addition, stock markets in Argentina, France, Germany, Greece, Italy, and Spain showed a 100% chance of being affected by COVID‐19 at some time. The WHO report of over 100 000 new daily cases of COVID‐19 worldwide in May 2020 (shaded area) led to fear, causing an increasing crisis severity index trend in some countries (e.g., Greece and Korea).

**Figure 3:**
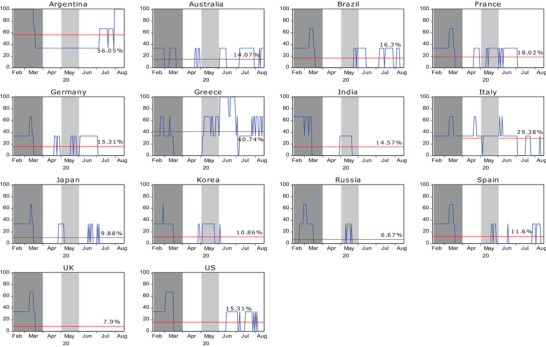
Crisis severity index for 14 countries during the COVID‐19 period using local currencies. Note: Crisis severity index (SI_*j*,*t*_(*i* → *j*)) is calculated in Equation (13) and unit is percentage (%). The horizontal line represents the mean of crisis severity index ($\mu_{{\text{SI}}_{j,t}}$).

Following the literature,^[^ ^11^, ^13^, ^32^ ^]^ this paper also adopts the local‐currency denominated returns for robustness checks and relevant results are presented in **Table** **3**. The results show that the empirical results using common currency denominated returns are robust to the results using local currencies. In Table 3, significant contagion effects in global equity markets can be identified from China to almost all countries in the other four regions, either through the cokurtosis or covolatility channels. The results are very similar to those in Table 2 except for Russia, which does not receive any contagion through the three channels when using local currencies. The possible reason is that the ruble depreciated by around 11% in March 2020 compared to the level in the beginning of the year due to the sharp fall in international oil prices.^[^ ^33^ ^]^ Regardless of whether the market rate of return is denominated in a common currency or a local currency, the contagion effects of China's equity market on other countries’ markets after the pandemic outbreak are mainly through the cokurtosis channel. **Figure** **3** shows the crisis severity index for the 14 countries using the local currencies. After changing the currency in which the rate of return is denominated, the trend of the severity index during the pandemic is almost the same. This feature is particularly obvious in the dynamic results of Latin American countries such as Argentina and Brazil.

**Table 3:** Test statistics for contagion based on changes in cokurtosis and covolatility during the COVID‐19 pandemic using local currencies

Country^a)^ (*j*)	Contagion tests					
	CK_13_	*p*	CK_31_	*p*	CV_22_	*p*
Asia						
Australia	35.73	0.00**	6.13	0.01**	0.15	0.70
India	31.55	0.00**	0.38	0.54	0.21	0.65
Japan	0.47	0.49	2.71	0.10	0.64	0.42
Korea	1.07	0.30	14.06	0.00**	7.02	0.01**
Europe						
France	13.56	0.00**	18.19	0.00**	0.24	0.63
Germany	8.12	0.00**	13.79	0.00**	0.91	0.34
Greece	41.99	0.00**	3.71	0.05**	0.38	0.54
Italy	94.73	0.00**	38.39	0.00**	0.23	0.63
Russia	1.99	0.16	2.24	0.13	0.17	0.68
Spain	25.88	0.00**	12.84	0.00**	0.04	0.84
UK	21.83	0.00**	4.13	0.04**	0.37	0.54
Latin America						
Argentina	934.96	0.00**	2.44	0.12	14.11	0.00**
Brazil	63.45	0.00**	1.34	0.25	0.54	0.46
North America						
US	20.54	0.00**	5.93	0.01**	0.38	0.54

^a)^ CK_13_: cokurtosis contagion test in Equation (4); CK_31_: cokurtosis contagion test in Equation (5); CV_22_: covolatility contagion test in Equation (9). *p = 0.1, **p = 0.05.

**Figure 4:**
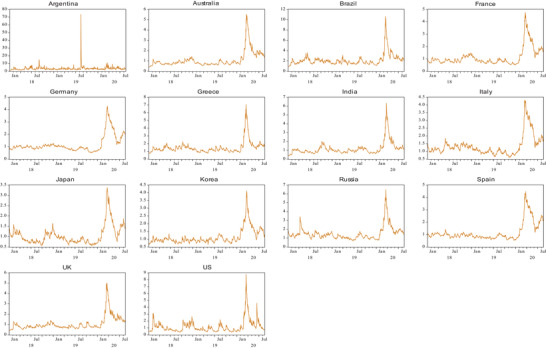
Conditional volatility (standard deviation) for 14 countries during the COVID‐19 period using US dollars.

In order to compare the results of contagion tests with the traditional time series model, this paper also uses the EGARCH conditional variance model and relevant results are presented in **Table** **4** and the conditional volatility of each index is plotted in **Figure** **4**. With regard to the mean contagion coefficients (*b*_2_), Asian, North American, and some Latin American countries have presented increased transmission mechanisms, while all European countries have presented decreased transmission mechanisms. However, no coefficients are significant at any significance level. With regard to the volatility contagion (parameter *d*_2_), it shows that all countries, except for India, Argentina, and the United States, have been affected by significant volatility contagion. Specifically, the 11 countries with significant volatility contagion effect have experienced increased volatility during the pandemic with positive values of the parameters of *d*_2_. Therefore, the conclusions based on the EGARCH model are consistent with the empirical results obtained from the previous extremal dependence tests, that is, there is significant contagion from China's equity market to the European countries, Asian countries (Australia and South Korea), and Brazil. As shown in Figure 4, the trends of volatility fluctuations of the market returns of 14 countries are similar within the entire sample period. While Argentina experienced significant changes in August 2019 due to the primary election and sharp fall in its stock market,^[^ ^34^ ^]^ the market performance of all countries fluctuated sharply from January to February 2020, corresponding to the pandemic.

**Table 4:** Empirical results for the EGARCH conditional variance model

	*c*	γ	θ	δ	*µ*_2_	*b*_1_ ^a)^	*b*_2_	*d*_1_	*d*_2_
Asia									
Australia	−0.108	0.144	−0.085	0.974	−0.002	0.218	0.047	−0.006	0.016
	0.000***	0.000***	0.000***	0.000***	0.956	0.000***	0.487	0.053*	0.001***
India	−0.110	0.138	−0.115	0.975	−0.028	0.186	0.909	0.005	0.006
	0.000***	0.000***	0.000***	0.000***	0.404	0.000***	0.170	0.138	0.141
Japan	−0.034	0.042	−0.112	0.981	−0.009	0.249	0.347	−0.002	0.011
	0.046**	0.03**	0.000***	0.000***	0.799	0.000***	0.537	0.457	0.000***
Korea	0.009	0.018	−0.117	0.972	−0.056	0.413	0.017	−0.015	0.021
	0.623	0.443	0.000***	0.000***	0.170	0.000***	0.780	0.000***	0.000***
Europe									
France	−0.010	0.008	−0.123	0.986	−0.006	0.219	−0.038	−0.001	0.011
	0.417	0.567	0.000***	0.000***	0.879	0.000***	0.460	0.590	0.000***
Germany	0.024	−0.037	−0.089	0.985	0.016	0.254	−0.063	−0.002	0.015
	0.060*	0.024**	0.000***	0.000***	0.648	0.000***	0.256	0.252	0.000***
Greece	−0.107	0.161	−0.097	0.961	0.006	0.260	−0.015	0.003	0.010
	0.000***	0.000***	0.000***	0.000***	0.898	0.000***	0.860	0.573	0.037**
Italy	0.030	−0.033	−0.120	0.983	0.012	0.219	−0.060	−0.005	0.013
	0.000***	0.000***	0.000***	0.000***	0.621	0.000***	0.393	0.001***	0.000***
Russia	−0.020	0.064	−0.137	0.967	0.028	0.219	−0.060	−0.008	0.013
	0.341	0.041**	0.000***	0.000***	0.573	0.000***	0.608	0.017**	0.000***
Spain	0.020	−0.025	−0.116	0.983	−0.045	0.211	−0.060	−0.004	0.016
	0.109	0.112	0.000***	0.000***	0.206	0.000***	0.304	0.037**	0.000***
UK	−0.105	0.151	−0.107	0.968	−0.026	0.215	−0.012	−0.011	0.018
	0.000***	0.000***	0.000***	0.000***	0.430	0.000***	0.876	0.001**	0.001**
Latin America									
Argentina	−0.149	0.849	0.076	0.817	0.111	0.519	−0.128	−0.013	−0.005
	0.0130**	0.000***	0.0429**	0.000***	0.141	0.000***	0.155	0.146	0.728
Brazil	−0.075	0.213	−0.110	0.951	0.027	0.203	0.097	−0.016	0.017
	0.002***	0.000***	0.000***	0.000***	0.686	0.001***	0.397	0.009***	0.011**
North America									
US	−0.274	0.364	−0.167	0.957	0.043	0.124	0.066	0.000	−0.002
	0.000***	0.000***	0.000***	0.000***	0.147	0.000***	0.308	0.924	0.838

^a)^ *b*_1_: contagion effect in mean during the crisis period; *d*_2_: the contagion effect in volatility during the crisis period. *p = 0.1, **p = 0.05, ***p = 0.01.

## 5 Conclusion

By applying the extremal dependence tests of contagion developed by previous work,^[^ ^21^ ^]^ this paper investigated the impact of the COVID‐19 pandemic on global stock markets. Using longitudinal stock data (i.e., daily stock prices between 2018 and 2020) collected from 15 countries, this research shows that the COVID‐19 crisis has had a direct and significant impact on global stock markets in four regions. In particular, the Latin and North American regions are highly exposed to contagion risk, followed by Europe, with the lowest contagion risk in Asia. This result corresponds to the findings of other researchers. During the COVID‐19 pandemic, there is significant contagion effect from China's stock markets to other different countries’ markets, and the pattern of financial contagion is similar to that of epidemic trend.^[^ ^10^, ^35^ ^]^

For countries with more severe epidemics, there are stronger contagion effects. The possible reason is that when the epidemic became more serious in a specific country, the panic caused by the lockdown or the restriction on gatherings led to a drastic decline in investors’ expectations for the future economy, and finally led to a sharp decline and volatility in the relevant stock market.^[^ ^35^ ^]^ According to the statistics of the WHO, as of May 2021, the Americas is still the region with the highest number of infections, with more than 67 million people, followed by Europe, with more than 54 million people. The western pacific is the region with the least number of infections among all regions, with more than 3 million people.^[^ ^36^ ^]^ Therefore, based on our empirical results, the impacts of the financial contagion during the pandemic in these regions have gradually diminished in such an order.

This paper provides high empirical value for reviewing the contagion effect of the Covid‐19 pandemic. Based on extremal dependence tests, we not only found significant financial contagion during the pandemic but also compared the different levels of impacts on different regions. To deal with the exogenous definition of the precrisis and COVID‐19 periods, the crisis severity index was computed to measure the degree of sensitivity of markets to COVID‐19. Based on the time window of the crisis severity index, we found that Latin America is most likely to be affected. Therefore, for the governing authorities of various countries, if they want to prevent the contagion of financial crises during the pandemic, strong and timely epidemic prevention measures are very necessary. The methods that can be used for reference include a combination of early travel restrictions, large‐scale testing, contact tracing, and stringent quarantine measures. These methods helped Asian countries, such as Mongolia and Vietnam, achieve remarkable results in epidemic prevention.^[^ ^37^ ^]^ From the results of this paper, the Asian countries also have stronger characteristics to resist financial contagion during the pandemic. Furthermore, for the countries vulnerable to financial crises, special precautions can be taken against the financial risks brought about by the epidemic. Measures to maintain sufficient liquidity in the financial system such as postponing debt repayment, implementing stimulus plans and credit guarantees can be considered.^[^ ^38^ ^]^

## Conflict of Interest

The authors declare no conflict of interest.

## Data Availability

Research data are not shared.
